# Supplementary figures and images for: Tumor immune microenvironment permissive to metastatic progression of ING4-deficient breast cancer
Source: PLoS One. 2024 Jul 5;19(7):e0304194. doi: 10.1371/journal.pone.0304194 (PMC11226078; doi:10.1371/journal.pone.0304194)

**S1 Fig.** Uncropped images of Western blots in Fig 2B.

Nuclear fraction

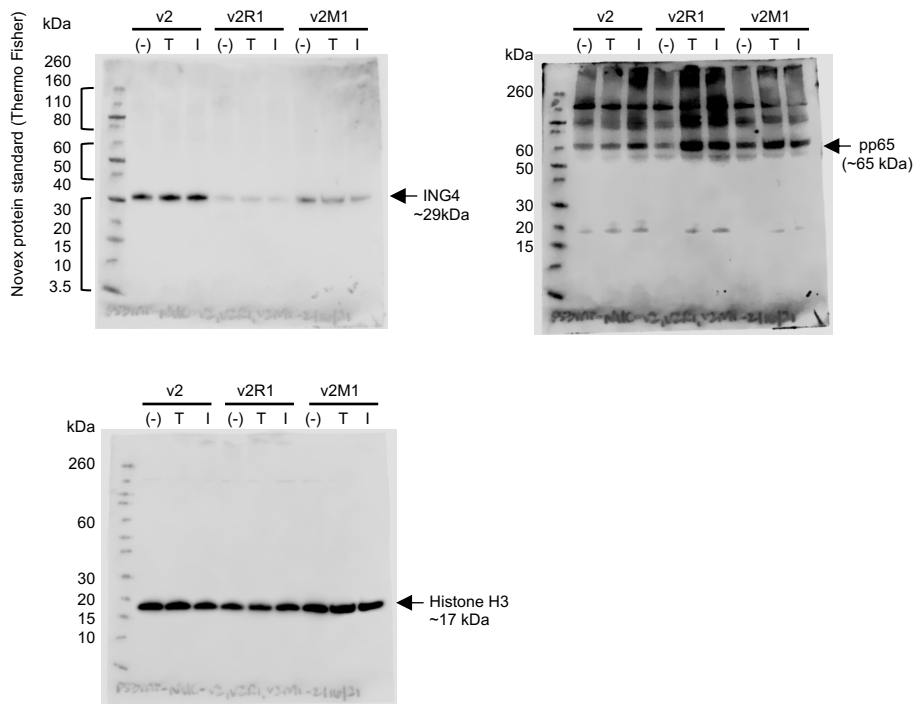

Cytoplasmic fraction

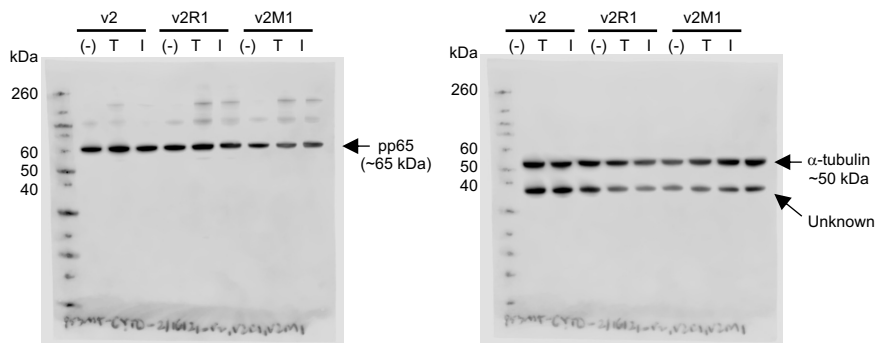

Supplement: S1 Fig — (PDF) [file pone.0304194.s001.pdf]
