# Supplementary material for: Tumor immune microenvironment permissive to metastatic progression of ING4-deficient breast cancer
Source: PLoS One. 2024 Jul 5;19(7):e0304194. doi: 10.1371/journal.pone.0304194 (PMC11226078; doi:10.1371/journal.pone.0304194)
Supplement: Attachment — Submitted filename: Tsutsumi et al Response to reviewer comments_050424.pdf [file pone.0304194.s002.pdf]

May 4, 2024

Dr. Emily Chenette, Ph.D.
Editor-in-Chief, *PLoS One*

Dear Dr. Chenette,

Thank you for your consideration to review our manuscript titled, "Tumor immune microenvironment permissive to metastatic progression of ING4-deficient breast cancer." This is a follow up submission to PONE-D-24-14191 and includes the corrections and revisions in response to the reviewer's comment.

First, we fully appreciated the instructive comments of the reviewer and are grateful for the opportunity to address the concerns. Below list the reviewer's comments (in black) and our responses (in blue).

Please let me know if you have any questions or additional concerns.

Sincerely,
Suwon Kim, Ph.D., the corresponding author

Reviewer's comments:

In your submission, you have not provided us with sufficient details regarding the animal research study design, rationale, methodology, or results to allow for internal evaluation of whether your study meets these standards and can be sent for peer review.

Specifically, we noted that the following details are missing from your Methods section: The number of animals used in the experiments, the frequency of animal monitoring, including the specific criteria you used to monitor animal health, animal welfare considerations such as postoperative care, the method of euthanasia, the methods of anesthesia or analgesia and other relevant steps taken to alleviate suffering or distress. The manuscript also lacks a specific, scientific, and ethical justification for allowing larger tumor sizes than is typically considered acceptable.

Response:

1) We added a subsection in the Methods section titled, "Mice" in pp6-7 lines 115-141, that included the information:
   - the frequency of animal monitoring,
   - the specific criteria to monitor animal health
   - the justification of the endpoint tumor size
   - animal welfare considerations including postoperative care, the method of euthanasia, anesthesia and optional analgesia
   - other relevant steps taken to alleviate suffering or distress.

2) We revised in the Methods section subtitled, "p53MT tumor implants and lung metastases in mice" in pp9-10 lines 183-194 that describes the mouse experiments in detail and lists:
   - the number of animals used in the experiments
   - the number of mice that were euthanized before the endpoint was reached as part of the effort to alleviate suffering.

3) We provided a scientific justification of the new mouse model of triple-negative breast cancer in p15 lines 343-357.

4) As for the tumor size measurement in Figure 3B, we had mislabeled the Y axis as “tumor diameter” where in fact, it was the “longest axis” measurement. The resected tumor “diameters” were within the IACUC approve side range ≤2-2.5cm. Justification of the tumor size range ≤2-2.5cm is not written in the Method section “Mice.” We corrected the Y-axis label in Figure 3B and revised the figure legend (p18, line 420) and the text (p17, line 406).
