# Supplementary material for: Tumor immune microenvironment permissive to metastatic progression of ING4-deficient breast cancer
Source: PLoS One. 2024 Jul 5;19(7):e0304194. doi: 10.1371/journal.pone.0304194 (PMC11226078; doi:10.1371/journal.pone.0304194)
Supplement: Attachment — Submitted filename: Tsutsumi et al Response to reviewers_061824.pdf [file pone.0304194.s003.pdf]

June 18, 2024

Dr. Emily Chenette, Ph.D.
Editor-in-Chief, *PLoS One*

Dear Dr. Chenette,

Thank you for the invitation to submit a revised version of the manuscript [PONE-D-24-17957] entitled, "Tumor immune microenvironment permissive to metastatic progression of ING4-deficient breast cancer." We also would like to thank the reviewers for their endorsement of our work and the manuscript. We also fully appreciate the instructive comments from the Reviewer #2. Below list the reviewer's comments (in black) and our responses (in blue).

Please let me know if you have any questions or additional concerns.

Sincerely,
Suwon Kim, Ph.D., the corresponding author

Reviewers' comments:

Reviewer #1: The manuscript submitted by Kim et al, explores the role of ING4 tumor suppressor gene in breast cancer. With a series of mouse experiments supported by analysis of METABRIC gene expression dataset, authors have deciphered the role of ING4 in breast cancer. The manuscript is well written and is easy to comprehend. Interpretation of this paper is well supported by the data. The role of ING4 in cancer in general has been hypothesized in previously published literature, and this work represents a considerable effort to address some of the open questions in this area of research. I recommend this paper to be published in it's current format.

We thank the reviewer for endorsing the merits of our work and recommending our manuscript for publication!

Reviewer #2: In the study entitled "Tumor immune microenvironment permissive to metastatic progression of ING4-deficient breast cancer" authors have made an excellent effort to understand the mechanism of how ING4-driven tumor-immune modulation creates an immune-evasive tumor microenvironment in breast cancer contributing to metastatic progression and poor patient survival.

But few concerns need attention to meet the publication standard of the journal.

We thank the reviewer for recognizing our work contribution to the field of ING4-deficient breast cancer. We are also grateful for the instructive suggestions to make the revised manuscript clearer, as listed below.

1. Materials and Methods section: please make separate subsections for each and every techniques used for ease of readability and better clarity. For instance, "Tumor Tissue Microarray (TMA) and Immunohistochemistry (IHC)" should be splitted into two separate sub-sections. Same to be followed for rest for the portions in this section where applicable.
We have separated the subsections in Methods as recommended. Separated subsections are: IHC (p.6), Quantification of lung metastases in mice (p.10), Flow cytometry (p.11), and Transwell migration assay (p.12).

2. The IHC images in Figure 1 are low resolution and pixelated. Please replace the same with high resolution clear images.
We replaced the images with high-resolution Tiff files instead of JPEG files used in the previous submission.

3. Please explain the 1-3 scoring system of IHC images. Is it done manually or using any specific software to eliminate the bias in scoring.
The IHC scoring was done manually by two trained personnel including a board-certified pathologist (J.L) who is a co-author on the paper. This is stated in the Methods (p.6). Two trained personnel scoring alleviated the bias in IHC scoring. We also added more detailed information on IHC staining of the TMAs (p.6). In addition, we added the individual IHC scores assigned to the images presented in Figure 1A to help visualize some of the scores.

4. Please include the images of western blots along with protein ladder in supplementary section.
The whole blot images with protein ladder were added as a supplementary material.

5. Please include the detailed and complete panel of flowcytometry data in supplementary section. the gating strategy is confusing.
We agree with the reviewer that the gating strategy in Figure 5A was not presented clearly. We have revised it to make it clearer and easier to understand. This revision, we believe, is sufficient to clarify the point.

6. Please remake figure 5 for better understandability. figure 5B is very confusing.
We revised Figure 5A to present the gating strategy clearly and labeled the x-axis of the bar graph in Figure 5B to represent the cell types yielded from the gating clearly. We also added sentences in the Figure legends of Fig 4 and Fig 5 to explain the bar graphs clearer.

7. Please include the foldchange readouts of the gene expression data for better clarity and conclusion.
The average fold changes were added as numbers on each bar in Figure 2C.

Additional Editor Comments:

Authors need to add clarity to the methodology and adress reviewers' comments regarding clarity of Gating strategy for flow cytometry in detail. I agree with the reviewer to furnish Western Blot images in the supplementary section. I encourage the authors to elaborate the method section as it is too condensed for readers comfort point of view.
Addressed as recommended (see above).
